# The influence of digital transformation on chinese bank profitability performance

**DOI:** 10.1371/journal.pone.0340249

**Published:** 2026-01-23

**Authors:** Senbo Zhang

**Affiliations:** 1 Boston University, Boston, Massachusetts, United States of America; An-Najah National University, PALESTINE, STATE OF

## Abstract

This paper aims to investigate the impact mechanisms of digital transformation in the banking sector under the rise of financial technology. Utilizing panel data from 116 commercial banks in China from 2011 to 2023, this empirical research employs regression analysis to explore how digital transformation in commercial banks can affect profitability performance. The findings reveal several key points: First, the implementation of digital transformation has not led to improvements in the profitability performance of most commercial banks; rather, it appears to reduce net interest margins and return on assets. Second, digital transformation primarily influences profitability performance through two bridging pathways—operational efficiency and non-performing loan ratio, with both playing a mediating role. Third, digital transformation and inclusive digital finance interact, affecting the profitability performance of banks, where the loan-to-deposit ratio plays a significant role. The heterogeneity of regional inclusive digital finance and banks’ loan-to-deposit ratios also influences the impact of corporate digital transformation on profitability performance. This research not only deepens the theoretical understanding of how digital transformation can help banks enhance their profitability performance but also provides valuable insights and recommendations for managers and operators in the banking sector during the digital transformation process.

## 1. Introduction

Since the dawn of the 21st century, advancements in big data and information technologies—such as artificial intelligence, blockchain, online conferencing, and e-commerce—have profoundly impacted the globe, transforming and even overturning traditional modes of production and daily life. The construction of the digital economy has become a global strategic priority. According to the White Paper on Global Digital Economy (2022) [1], the value added by the digital economy in 47 major countries reached $38.1 trillion in 2021, with China contributing $7.1 trillion, ranking second only to the U.S. ($15.3 trillion). In recent years, the development of digital finance in China has been at the forefront globally [2], bringing significant changes to its financial system. As the backbone of this system, the banking sector has initiated a wave of digital transformation.

Initially, internet companies adopted digital finance technologies, which subsequently impacted banks’ operations in payment processing, wealth management, and lending. In response to the challenges posed by this digital wave, Chinese commercial banks have also embarked on digital transformation initiatives. Consequently, enterprises are actively or passively undergoing digitization in response to changes in digital technology, continuously adjusting their business models, development strategies, and management practices [3,4]. Particularly within traditional financial systems, an increasing number of commercial banks are recalibrating their development strategies and organizational structures to adapt to the disruptive changes brought about by financial technology. They are implementing top-down reforms to respond to competitive pressures and leveraging emerging digital technologies to enhance profitability performance [5]. Given that China’s financial system is dominated by the banking sector, which holds a unique and critical position, this paper explores the impact of digital transformation on bank performance, specifically focusing on profitability performance.

According to information disclosed by commercial banks, the six major state-owned banks in China invested a total of 116.549 billion yuan in financial technology in 2022, while the top ten joint-stock banks invested 64.768 billion yuan. In 2022, the technology investment of Bank of Communications grew by 32.93% year-on-year, while China Bank and Agricultural Bank of China experienced increases of 15.70% and 13.05%, respectively. Beginning in 2019, major banks have fully disclosed their technology strategies, investments, and personnel in their annual reports, reflecting the intensity of digital transformation efforts within the Chinese banking sector. Research indicates that banks’ engagement with financial technology can enhance efficiency through information and communication innovations [6]. However, other studies have found no significant positive correlation between digital technology and corporate performance, suggesting that only certain enterprises benefit from digitization [7]. Data released by national financial regulatory authorities show that the net profit of Chinese commercial banks rose to 2.303 trillion yuan in 2022, an increase of 5.4% year-on-year, although profit growth has slowed compared to previous years. The total amount of non-performing loans reached 2.98 trillion yuan, with a year-on-year growth rate of 4.8%, maintaining a low growth trend.

The digital transformation of banks ensures a pragmatic process from top-level design to grassroots implementation, which is crucial for achieving the goals of enhancing bank profitability. Existing literature presents differing conclusions regarding the relationship between digital transformation and profitability performance: digital transformation can significantly increase total factor productivity but may also decrease firm performance by raising operational costs, reducing total asset turnover, and increasing management expenses [8]. A U-shaped relationship exists between digital transformation and bank profitability; Digital transformation supports long-term profitability, it may lead to short-term profitability deterioration due to substantial IT investments [9]. Conversely, other studies indicate that digital talent and capabilities positively and significantly influence bank performance [10], and that digital banks with a higher number of customers and transactions via digital communication perform better [11]. These contradictory conclusions prompt critical reflection; Thus, the first question this paper seeks to explore is: How does the digital transformation of banks impact profitability performance?

Different perspectives in the literature investigate the mechanisms through which bank digital transformation influences profitability performance, particularly concerning operational efficiency and bad debt ratios. In terms of liability impact, financial institutions with significant digital transformation investments prioritize lending to higher-risk borrowers [12]. While this may lower borrowing costs in the short term, it can ultimately plunge enterprises into debt crises, increasing the risk of default [13]. However, from a regulatory standpoint, authorities leveraging financial technology and risk management can more efficiently monitor repayment records and cash flow [14], thereby mitigating fraud and default risks, which in turn can reduce banks’ bad debt ratios. As net interest income primarily derives from traditional deposit and loan operations, banks that overly depend on customer interest income may increase output by expanding branch networks and raising fixed asset and employee counts, negatively impacting cost efficiency [15]. Digital transformation, however, can help commercial banks reduce their reliance on asset-liability scale expansion, thus stabilizing income and improving efficiency. This leads us to the second question of this paper: Are operational efficiency and bad debt ratios mediating variables in the relationship between the digital transformation in banks and profitability performance?

Additionally, literature has explored the heterogeneous effects of external digitalization and internal loan-to-deposit ratios on the impact of bank digital transformation on profitability performance. Digital inclusive finance increases the supply of financial products and services, effectively broadening the coverage of financial services [16]. While enhanced financial inclusiveness leverages banks’ informational advantages, it may also exacerbate agency problems among corporate management, leading to short-sighted decisions and overestimations of future projects [17]. The development of digital inclusive finance deepens access to financial services, with technologies like big data and artificial intelligence improving banks’ service efficiency and aiding in addressing issues like information asymmetry and risk identification [18]. An increase in the level of digital finance effectively resolves the “disconnection from reality” problem associated with traditional credit disbursement, thereby enhancing credit efficiency [19]. The loan-to-deposit ratio is closely related to the asset-liability structure, and a relatively healthy state is typically maintained within a certain range. When commercial banks pursue profit maximization without restraint, they often opt to increase lending and gradually shift toward lending to borrowers with lower credit ratings. This approach directly affects credit costs, influencing the repayment ability of borrowing enterprises, reducing the quality of bank loans, and resulting in a rise in non-performing loans, which in turn impacts profitability performance [20]. Hence, the third question this paper seeks to address is: are external digitalization levels and internal loan-to-deposit ratios boundary conditions in the relationship between bank digital transformation and profitability performance?

Based on these three questions, this paper employs panel data regression analysis, utilizing micro-level panel data from 116 commercial banks from 2011 to 2023 to examine the impact of digital transformation on enhancing quality and efficiency in banking. The marginal contributions of this paper are threefold: First, it conducts a longitudinal analysis of digital technology in the banking sector, addressing the “Productivity Paradox”—the apparent disconnect between corporate IT investment and returns—thereby helping commercial banks adjust their expectations regarding digital transformation from a theoretical perspective. Second, it reveals operational efficiency and bad debt ratios as two critical mediating variables, elucidating their roles in the mediating influence mechanism. Third, it provides a heterogeneous analysis of the national inclusive finance index and loan-to-deposit ratios, innovatively discussing how the digital inclusive finance environment and loan-to-deposit ratios affect the impact of bank digital transformation on profitability performance. The conclusions of this paper systematically analyze and discuss the mechanisms through which digital transformation influences quality enhancement and efficiency in the banking sector, offering significant theoretical contributions and practical insights for the industry.

## 2. Literature review and research hypotheses

### 2.1 Conceptual definitions

#### (1) Digital transformation in banking.

According to a 2021 survey by the World Bank, digital transformation has become a strategic priority for the majority of traditional commercial banks [21]. Developments in information technology—such as continuous connectivity, social networks, and the Internet of Things, combined with artificial intelligence—are reshaping society [22]. In the context of commercial banks, digital transformation refers to the integration of digital technologies across all areas of banking, fundamentally altering how these institutions operate and deliver value to customers. This includes the implementation of financial and banking software, digital banking platforms, mobile banking solutions, and fintech services, all aimed at meeting customer demands related to interest rate liberalization, big data, mobile finance, risk management, internet finance, and customer relationship management [11,23]. Digital transformation plays a crucial role in reshaping traditional interactions between customers and banks [24].

Research on digital transformation in banks can be categorized into four main areas. First, from an organizational perspective, studies examine the impacts of digital transformation on operational management [25] and departmental collaboration [26]. Second, from a digital technology perspective, investigations explore how banks utilize digital technologies to enhance competitiveness [27] and innovate within traditional business applications [28]. Third, focusing on the methods of digital transformation, research analyzes customer data in banks based on big data [29] and the development of fintech through investment [30] and external collaboration [31]. Fourth, from the perspective of operational services, studies discuss the effects of digital transformation on operational efficiency, and risk management [32].

The digital transformation of banks encompasses three key dimensions: digital readiness, customer centricity, and digital leadership, which collectively aim to simplify banking services for customers [33]. Barroso and Laborda [34] emphasizes that digital transformation in the financial sector is largely driven by advancements in financial technology, which provide innovative solutions to traditional banking practices and enable the development of new financial services. Nwoke [35] argues that digital transformation redefines the organizational culture and customer engagement models within banks, with fintech playing a central role in this transition. Zhang et al. [36] highlights that financial technology is an essential enabler for digital changes in financial institutions, particularly in enhancing the speed and scope of digital transformation in banks.

Current literature on bank digital transformation predominantly highlights its positive impacts, while research on its negative effects remains limited. Therefore, this paper analyzes data from the Chinese banking sector between 2011 and 2023 to investigate the effects and mechanisms of digital transformation on profitability performance. It is hoped that this research will expand understanding of digital transformation in banking and offer a more objective perspective for the public to critically evaluate this phenomenon. Utilizing operational efficiency and bad debt ratios, this paper indirectly measures the impact of digital transformation on profitability performance in banks, thereby enriching the discourse on the pathways through which digital transformation affects banking profitability performance.

#### (2) Profitability performance in banking.

The profitability performance of commercial banks reflects their ability to generate sustainable earnings and create value for shareholders. According to Dietrich and Wanzenried [37], profitability is commonly evaluated using indicators such as return on assets (ROA), return on equity (ROE), and net interest margin (NIM), with ROA being a particularly important measure because it reflects the ability of a bank’s management to generate profits from the bank’s assets. Golin and Delhaise [38] also points out, the ROA has emerged as the key ratio for the evaluation of bank proﬁtability and has become the most common measure of bank proﬁtability.

Research has shown that financial technology can enhance bank profitability by expanding customer reach, improving product personalization, and reducing costs associated with traditional service delivery channels [39]. Existing studies generally demonstrate that financial technology has positive economic effects [40].

Compared with existing studies, this research offers a more nuanced understanding of the impact of digital transformation on banks’ profitability performance. While prior literature has predominantly examined either financial outcomes or broad technological adoption, this paper focuses on how digital transformation improves profitability through changes in loan structure and operational efficiency, treating these as key channels through which performance outcomes are realized. Furthermore, by introducing the Inclusive Finance Digital Index as a moderating variable, this paper extends the analytical framework and reveals how varying levels of digital inclusiveness influence the effectiveness of digital transformation. These contributions help clarify the underlying mechanisms through which digital transformation affects bank profitability performance and provide practical implications for banks seeking to optimize their digital strategies.

### 2.2 Theoretical framework and research hypotheses

#### (1) The impact of digital transformation in banking on profitability performance.

From an organizational perspective, digital transformation often involves large-scale structural changes and process redesigns. The adoption of digital transformation can trigger adjustment costs, staff training expenses, and resource reallocations that suppress profitability ratios, including ROA [41]. Such frictions can reduce income streams, when significant managerial and financial resources are diverted from revenue-generating activities to digital projects [42].

From a strategic investment perspective, large-scale digital transformation programs require substantial upfront capital—including IT infrastructure upgrades, business system integration, and workforce training [30]. These investments typically create considerable cost pressures before the revenue benefits are realized [43]. The time lag between cost incurrence and benefit realization aligns with the “digital paradox” described by Gebauer et al. [44], wherein financial returns from digital investments often fall short of expectations during the initial stages, with this negative impact being directly reflected in ROA [30].

Furthermore, digital transformation can change the business structure and customer composition of banks, resulting in temporary fluctuations in interest margins, fee income, and risk exposure [45]. These fluctuations can further influence the performance of ROA.

In summary, during the resource investment and implementation phases, the combined effects of high capital expenditure, organizational adjustment costs, and business structure fluctuations are likely to cause a decline in ROA.

Based on this analysis, the following hypothesis is proposed:

Hypothesis 1: Digital transformation in banks negatively affects the return on assets.

#### (2) The mediating role of operational efficiency and non-performing loan ratio.

Further research can explore the relationship between operational efficiency and non-performing loan ratios, particularly in the context of credit activities. During the resource-investment and transition phase of digital transformation, in response to the pressures of digital finance and to maintain competitiveness, small and medium-sized banks often set ambitious transformation objectives and commit substantial human and material resources, which can elevate coordination costs, induce process frictions, and reduce operational efficiency [6]. Recent studies highlight the so-called productivity/digital paradox, whereby digital transformation can hinder efficiency improvements due to integration complexity, learning costs, and organizational frictions, thereby weighing on profitability metrics such as ROA [9]. Empirical evidence confirms that the bank profitability performance (ROA/ROE) can even be reduced during the transformation stages[46].

Lending activities represent a core function of commercial banks, and information asymmetry between borrowers and lenders during the credit decision-making process is a critical factor contributing to banking risks [47]. Wójcik-Mazur and Szajt [48] reveal that a lower non-performing loan ratio is a crucial determinant of profitability in EU banks, suggesting that “efficient credit risk management is not only important for stability but also for sustaining high ROA levels”.While digital transformation can enhance data collection and automation, during the adoption phase it may also raise banks’ risk-taking, relax screening standards, and expand lending to weaker borrower segments, thereby increasing non-performing loans. Evidence from China shows that digital transformation can elevate credit risk, particularly when risk control mechanisms are underdeveloped [49,50], while studies also report increases in loan loss provisions due to adjustment frictions and aggressive loan expansion [51]. Similar findings are observed in Vietnam, where digital transformation investments temporarily raise credit risk [52], and in Europe, where digital transformation phases are linked to non-performing loan increases caused by digital infrastructure gaps and operational misalignment [53].

Against this backdrop, the dual mediation is theoretically coherent in the short to medium run: (i) Digital transformation-induced coordination, integration, and learning costs reduce operational efficiency, thereby dragging ROA; (ii) Digital transformation-enabled expansion and competitive pressure can raise risk-taking and push up non-performing loans, which further depress ROA. This clarifies why digital transformation impacts can be negative via operational efficiency and non-performing loan channels during the transition.

Based on this analysis, the following hypotheses are proposed:

Hypothesis 2: Digital transformation in banks negatively affects the return on assets by reducing operational efficiency.

Hypothesis 3: Digital transformation in banks negatively affects the return on assets by increasing the non-performing loan ratio.

## 3 Research design and variable model

### 3.1 Research sample and data sources

This paper analyzes Chinese commercial banks with complete and consistent public disclosures over 2011–2023, using the Digital Transformation Index for commercial banks constructed by the Digital Finance Research Center at Peking University [54]. Annual financial data for the banks is obtained from the Guotai An database and the Wind database, with missing values supplemented through manual collection of annual reports disclosed by the banks. Regional data is sourced from the “China City Statistical Yearbook” and the National Bureau of Statistics website. After excluding samples with missing variables, a panel dataset consisting of 1219 observations from 116 banks across various provinces is obtained, covering large commercial banks, joint-stock commercial banks, urban commercial banks, and smaller rural commercial banks. Specific definitions of main variables are detailed in Table 1, with descriptive statistics provided in Table 2.

**Table 1:** Variable description.

Variable Type	Variable Name	Variable Symbol	Variable Design
**Dependent Variable**	Return on Assets	roa	Net profit/Average Total assets
**Core Independent Variable**	Digital Transformation	DT	Constructed from three dimensions: Strategic Digitalization, Business Digitalization, and Management Digitalization
**Mediating Variables**	Operating Efficiency	oe	Operating Income/ Operating Expenses
	Non-Performing Loan Ratio	nplr	Annual Non-Performing Loans/ Annual Credit Sales
**Control Variables**	Logarithm of Bank Asset Size	size	Logarithmic value of Total Bank Assets
	Loan-to-Deposit Ratio	LDR	Total Loans/ Total Deposits
	Provincial Economic Growth Rate	ProGDPr	GDP Growth Rate of the Province where the Bank is Located (%)
	Deposit Share	TD	Total Deposits/ GDP of the Province
	Loan Share	TL	Total Loans/ GDP of the Province
	Capital Adequacy Ratio	cra	Total Capital/ Risk-Weighted Assets
	Provision Ratio	pr	Loan Impairment Provisions/ Total Loans

**Table 2:** Summary statistics.

VarName	Obs	Mean	SD	Min	Median	Max
roa	1219	0.897	0.334	0.007	0.894	2.228
DT	1219	6.867	4.057	0.000	6.290	17.399
oe	1219	0.365	0.303	0.003	0.480	0.930
nplr	1219	0.014	0.006	0.000	0.014	0.037
size	1219	26.475	1.576	23.656	26.151	30.934
LDR	1219	0.685	0.119	0.346	0.682	1.000
ProGDPr	1219	7.590	1.977	1.200	7.600	13.600
TD	1219	2.244	1.070	1.200	1.880	5.190
TL	1219	1.567	0.453	0.840	1.410	2.530
cra	1219	0.135	0.021	0.105	0.132	0.254
pr	1219	2.619	2.069	0.000	2.114	18.911

### 3.2 Variable design

#### (1) Dependent variable.

According to Almaskati [55], bank profitability performance is commonly evaluated using financial indicators including the return on assets (ROA), return on equity (ROE), and net interest margin (NIM). Caballero et al. [56] argue that NIM offers only a partial view of bank performance, especially under changing market conditions, whereas ROA captures the full picture. Given that ROE can be significantly influenced by capital structure—potentially leading to inflated values in cases of inadequate equity capital [57]—and ROA is less affected by differences in leverage and capital structure than ROE, making it a more consistent measure of profitability across banks with varying capital bases [42], so the return on assets (ROA) is selected as the dependent variable.

#### (2) Core independent variable.

The core independent variable is the Digital Transformation Index of commercial banks, also sourced from the Digital Finance Research Center at Peking University [54]. This index is constructed from three dimensions: strategic digitalization, business digitalization, and management digitalization, providing a comprehensive and objective measurement of the digital transformation status and development trends of Chinese commercial banks.

#### (3) Mediating variables.

Financial innovations driven by digital transformation, such as electronic banking and online loans, reshape service delivery channels and business processes, thereby influencing the operational efficiency of commercial banks [58]. Digital transformation influences internal operations and organizational processes, thereby shaping firm-level productivity and return outcomes. This paper selects the ratio of operating income to operating expenses as a measure of bank operational efficiency, serving as a mediating variable. Additionally, digital transformation reduces the information asymmetry between banks and enterprises and optimizing traditional financial operating models [59]. Digital transformation has been shown to influence bank risk profiles, especially in managing credit risk, which is directly linked to non-performing loans. Digital transformation affects risk screening and portfolio quality, with evidence from China suggesting that the adoption of digital transformation has notable implications for the non-performing loan ratios of commercial banks. Mandagie [60] reports that higher non-performing loan ratios are associated with significantly lower ROA in banks listed on the stock exchange. Consequently, this paper includes the non-performing loan ratio as another mediating variable, as digital transformation can influence risk management efficiency and credit decision-making processes, thereby affecting profitability performance.

#### (4) Control variables.

Based on existing research [32,61,62], this paper incorporates additional control variables that influence bank profitability performance, including: the logarithm of bank asset size (size), measured as the logarithmic value of total bank assets; The loan-to-deposit ratio (LDR), calculated as the ratio of total deposits to total loans; the economic growth rate of the province where the bank is located (ProGDPr), measured by the GDP growth rate of that province; and the proportions of total deposits and loans relative to the province’s GDP (TD and TL), derived from the ratio of total deposits and loans to the GDP of the respective province. Additionally, capital adequacy ratio (cra) and provision ratio (pr) are included as control variables.

#### (5) Descriptive statistics analysis.

Table 2 presents the descriptive statistics of key variables, with all variables having 1219 observations, ensuring comparability.

ROA (Return on Assets) has a mean of 0.897 and a standard deviation of 0.334, showing relatively low volatility. DT has a mean of 6.867 and a high standard deviation of 4.057, with a maximum value of 17.399, suggesting significant differences. OE (Operating Efficiency) has a mean of 0.365 and a standard deviation of 0.303, with a maximum value of 0.930. NPLR (Non-Performing Loan Ratio) has a low mean of 0.014 and minimal fluctuations, indicating overall stable credit quality. Size (Firm Size) has a mean of 26.475 and a standard deviation of 1.576, suggesting firms are relatively similar in size. LDR (Loan-to-Deposit Ratio) has a mean of 0.685 and a standard deviation of 0.119, with little variation. ProGDPr (Proxy for GDP Growth Rate) has a mean of 7.590 and a standard deviation of 1.977, indicating some economic growth fluctuations. TD (Total Debt) and TL (Total Liabilities) also show moderate variations. PR (Profitability Ratio) has a mean of 2.619 but a high standard deviation of 2.069, with a maximum of 18.911, reflecting significant differences in firms’ profitability.

Overall, some variables (such as DT and pr) exhibit high variability, while others (such as nplr and LDR) remain relatively stable, highlighting the heterogeneity in firms’ financial conditions.

### 3.3 Model design

According to the research purpose and theoretical hypothesis of this paper, we construct the following fixed effect model to investigate the impact of bank digitization on return on assets. Based on the results of the Hausman test, a two-way fixed effects model is adopted. The econometric model is set as follows:


$$\begin{array}{c} {RoA}_{it}=\alpha_{\text{0}}+\alpha_{\text{1}}{DT}_{it}+\varphi C_{it}+{FE}_{firm}+{FE}_{year}+\varepsilon_{it}; \end{array}$$ (1)

Where, *i* represents the bank and *t* represents the year. The explained variable *RoA* represents the operating performance-return on assets, and the explanatory variable *DT* represents the overall digitization index. *C* represents the control variables.${FE}_{firm}$,${FE}_{year}$represent bank and year fixed effects, respectively. This paper controls the above two fixed effects to absorb the omitted effects. In addition, robust standard errors are clustered to the id-year level in all regressions.

### 3.4 Regression results and data analysis

#### (1) The impact of digital transformation on bank profitability performance.

Table 3 presents the regression results on the effect of digital transformation on bank profitability performance. Column (1) shows that, without control variables and without applying time or individual fixed effects, digital transformation has a significantly negative impact on return on assets (p < 0.01). Column (2) reveals that, when controlling for time fixed effects but without control variables, digital transformation still exerts a significantly negative influence on return on assets (p < 0.05). Column (3) demonstrates that, after controlling for both time and individual fixed effects but without any control variables, the impact of digital transformation on return on assets remains significantly negative (p < 0.05).

**Table 3:** Regression analysis.

	(1)	(2)	(3)	(4)	(5)
	roa	roa	roa	roa	roa
DT	−0.036^***^	−0.015^**^	−0.017^**^	−0.010^***^	−0.009^**^
	(0.001)	(0.002)	(0.003)	(0.003)	(0.003)
size				0.012	0.021
				(0.026)	(0.024)
LDR				−0.396^**^	−0.274^***^
				(0.102)	(0.103)
ProGDPr				0.034^***^	0.024^***^
				(0.005)	(0.006)
TD					−0.027
					(0.036)
TL					−0.227^***^
					(0.067)
cra					−0.482^*^
					(0.246)
pr					0.012^***^
					(0.004)
_cons	1.138^***^	1.117^***^	1.126^***^	0.736	0.928
	(0.029)	(0.029)	(0.021)	(0.677)	(0.623)
*N*	1219	1219	1219	1219	1219
R^2^			0.516	0.586	0.605
Year		Yes	Yes	Yes	Yes
Id			Yes	Yes	Yes

Standard errors in parentheses.

* *p* < 0.10, ** *p* < 0.05, *** *p* < 0.01.

In Column (4), after introducing control variables such as the logarithm of bank asset size, loan-to-deposit ratio, and the GDP growth rate of the bank’s province, while still controlling for time and individual fixed effects, digital transformation continues to have a significantly negative effect on return on assets (p < 0.01). Finally, in Column (5), after incorporating all control variables and continuing to control for time and individual fixed effects, the negative impact of digital transformation on return on assets persists as significant (p < 0.05).

These regression results provide robust support for Hypothesis 1, indicating that the digital transformation index has a significantly negative impact on return on assets. This suggests that digital transformation does not enhance bank profitability performance and, in fact, hinders it, thereby confirming the validity of Hypothesis 1.

#### (2) The mediating effect of digital transformation on bank profitability performance.

To address the mediation mechanism between digital transformation and bank performance, this paper follows the three-step causal approach proposed by Wen and Ye [63], an adaptation of the classical Baron and Kenny (1986) mediation framework [64]. This method involves (1) confirming the significance of the total effect of digital transformation on return on assets (ROA)—this condition has already been verified in the baseline regression analysis in Section 3.4 (1); (2) testing whether digital transformation significantly affects the mediating variables—operational efficiency and the non-performing loan ratio; and (3) examining whether these mediators significantly influence ROA while controlling for digital transformation.

Columns (1) to (4) of Table 4 report the results of the mediating effect tests of digital transformation on bank profitability performance, and also test the baseline regression results of Hypotheses 2 and 3. Columns (1) and (2) show that the coefficient of digital transformation on the mediating variable operational efficiency is significantly negative (P < 0.01), and the coefficient of operational efficiency on the return on assets is also significantly positive (P < 0.01). Columns (3) and (4) demonstrate that the coefficient of digital transformation on the mediating variable non-performing loan ratio is significantly positive (P < 0.01), while the coefficient of the non-performing loan ratio on return on assets is significantly negative (P < 0.01).

**Table 4:** Mediating effect.

	(1)	(2)	(3)	(4)
	oe	roa	nplr	roa
DT	−0.931^***^	−0.008^***^	0.025^***^	−0.007^***^
	(0.275)	(0.003)	(0.009)	(0.002)
oe		0.001^***^		
		(0.000)		
nplr				−0.062^***^
				(0.016)
size	−15.734^***^	0.034	0.005	0.021
	(2.753)	(0.025)	(0.097)	(0.024)
LDR	−0.708	−0.274^***^	0.572^**^	−0.239^**^
	(7.837)	(0.103)	(0.248)	(0.106)
ProGDPr	−0.966^**^	0.025^***^	0.015	0.025^***^
	(0.477)	(0.006)	(0.013)	(0.006)
TD	−2.579	−0.024^*^	0.006	−0.026
	(2.770)	(0.036)	(0.091)	(0.034)
TL	−2.767	−0.224^***^	−0.135	−0.235^***^
	(5.335)	(0.067)	(0.164)	(0.066)
cra	−9.643	−0.474^*^	−1.511	−0.576^**^
	(32.256)	(0.243)	(1.246)	(0.251)
pr	0.075	0.012^***^	−0.140^***^	0.003
	(0.359)	(0.004)	(0.019)	(0.004)
_cons	475.807^***^	0.534	1.036	0.993
	(75.767)	(0.659)	(2.636)	(0.620)
*N*	1219	1219	1219	1219
R^2^	0.831	0.608	0.468	0.619
Year	YES	YES	YES	YES
Id	YES	YES	YES	YES

Standard errors in parentheses.

* *p* < 0.10, ** *p* < 0.05, *** *p* < 0.01.

These regression results indicate that digital transformation in banks negatively impacts operational efficiency while increasing the non-performing loan ratio. Operational efficiency and the non-performing loan ratio act as mediators in the relationship between digital transformation and return on assets. In other words, digital transformation affects return on assets negatively by influencing both operational efficiency and non-performing loan ratio. Therefore, Hypotheses 2 and 3 are supported.

In the regression analysis, it is evident that digital transformation negatively impacts operational efficiency in banks. However, it is worth noting that this analysis uses data from 2011 to 2023. Despite the rapid development of digitalization and fintech in Chinese cities and enterprises during this period, this growth was not sufficient to enhance operational efficiency in banks. Major reforms in the banking sector are often driven top-down, requiring substantial policy, financial, and human resources. The understanding and implementation of digital transformation across different levels, shaped by long-standing business habits and operational models, require a significant adaptation period to realize the desired improvements in efficiency.

In the process of implementing digital transformation policies, banks face challenges in ensuring effective execution at the branch and sub-branch levels. For instance, setting representative performance indicators that can effectively measure the progress and implementation of digital transformation at the grassroots level remains a critical issue. Nonetheless, digital transformation has already been incorporated into the strategic development plans of major banks, and improvements in operational efficiency due to digital transformation in the coming decade are both optimistic and expected.

The regression results also show that digital transformation increases the non-performing loan ratio in banks, which suggests that the risk control capabilities of banks were weakened between 2011 and 2023. On the one hand, advancements in big data and fintech have driven banks towards digital transformation, but the technology has not yet developed to a stage where it can significantly help banks mitigate risks in real-time. On the other hand, under traditional operating models, banks used methods like on-site inspections to gather detailed and accurate information about borrowers’ financial status, loan purposes, and default probabilities. While digital transformation allows banks to shift pre-loan investigations and post-loan management online through tools like big data, artificial intelligence, and intelligent risk control systems, the amount of effective information available is still limited. Current technology has not yet evolved to the point where sufficient evaluation data or effective decision-making information can be obtained through purely digital means. Improving the accuracy of post-loan management and more timely access to a borrower’s financial updates are key areas where digital transformation still needs significant advancements.

## 4. Robustness test

To further verify the robustness of the regression results, two methods were employed. First, the independent variable was replaced. The second method replaced the independent variable. The digital transformation index was lagged by two periods (l2DT). As shown in Column (1) of Table 5, the regression result shows that the two-period lagged digital transformation index also has a significantly negative impact on return on assets, with a marginal effect of −0.007 (p < 0.01).

**Table 5:** Robustness test.

	(1)	(2)
	roa	roa
DT		−0.011^***^
		(0.003)
l2DT	−0.007^***^	
	(0.003)	
size	0.025	0.017
	(0.033)	(0.027)
LDR	−0.317^***^	−0.292^**^
	(0.103)	(0.120)
ProGDPr	0.021^***^	0.029^***^
	(0.006)	(0.009)
TD	−0.014	−0.033
	(0.037)	(0.044)
TL	−0.175^**^	−0.174^**^
	(0.070)	(0.086)
cra	−0.302	−0.501^*^
	(0.293)	(0.281)
pr	0.023^***^	0.007
	(0.005)	(0.005)
_cons	0.740	0.951
	(0.860)	(0.716)
*N*	1056	923
R^2^	0.561	0.598
Year	Yes	Yes
Id	Yes	Yes

Standard errors in parentheses.

* *p* < 0.10, ** *p* < 0.05, *** *p* < 0.01.

As a second robustness check, this paper addresses the potential systematic impact of the COVID-19 pandemic on bank profitability performance during the period 2020–2022. To mitigate this effect, the test excludes observations from these years and conducts regression analysis using only the data from the pre-pandemic (2018–2019) and post-pandemic (2023) periods. This approach allows us to examine whether the direction and statistical significance of the key explanatory variable remain consistent. As reported in Column (2) of Table 5, the negative effect of digital transformation on return on assets remains statistically significant even after excluding the pandemic years, with a marginal effect of −0.011(p < 0.01), thereby reinforcing the robustness of the study’s conclusions.

The digital transformation of commercial banks is a multifaceted process that extends beyond the adoption of fintech tools to include adjustments in business models and management practices. The robustness checks—using lagged variables and excluding pandemic years—consistently demonstrate that digital transformation has a significant negative impact on return on assets. This further confirms the robustness of the study’s conclusions.

## 5. Endogeneity test

The impact of digital transformation on bank profitability performance may be subject to three potential issues that could weaken the validity of the baseline regression results. First, there is the problem of reverse causality, where the bank’s own behavior may drive the digital transformation. Second, there is the issue of omitted variables, where despite controlling for regional economic development and individual characteristics of the bank, other factors may still influence both the digital transformation and the bank’s profitability performance. Third, there is the problem of spurious regression, as the dependent variable “return on assets”, the core independent variable “digital transformation index”, and some control variables like “logarithm of bank asset size” all show clear upward trends over time in line with economic development, which could lead to spurious regression results when regressing these variables directly.

To address the endogeneity caused by reverse causality and the spurious regression issue, this paper applies a lagged time series regression. Specifically, the lagged digital transformation index (lDT) of the next period is used as the explanatory variable to assess the impact of the following period’s digital transformation on the current period’s bank profitability performance. As shown in Column (1) and (2) of Table 6, the regression results show that the two-stage digital transformation index has a significant negative effect on return on assets. This finding is consistent with the baseline regression results, confirming the robustness of the study’s conclusions.

**Table 6:** Endogeneity analysis.

	(1)	(2)
	First	Second
lDT	0.502^***^	
	(0.033)	
DT		−0.014^***^
		(0.005)
size	0.357	0.039^*^
	(0.360)	(0.023)
LDR	2.466^***^	−0.278^***^
	(0.666)	(0.093)
ProGDPr	−0.107^***^	0.020^***^
	(0.037)	(0.005)
TD	−0.081	−0.018
	(0.360)	(0.030)
TL	−0.096	−0.232^***^
	(0.519)	(0.055)
cra	7.943^**^	−0.332
	(3.716)	(0.273)
pr	−0.015	0.015^***^
	(0.043)	(0.004)
_cons	−9.243	
	(9.455)	
*N*	1139	1139
R^2^	0.803	0.589
Year	Yes	Yes
Id	Yes	Yes

Standard errors in parentheses.

* *p* < 0.1, ** *p* < 0.05, *** *p* < 0.01.

## 6. Heterogeneity analysis

### 6.1 Heterogeneity of digital financial development environment

Digital financial development varies across regions where banks operate. To analyze the heterogeneity of digital financial development, this paper adopts the Digital Financial Inclusion Index, which has gained widespread academic recognition and was published by the Digital Finance Research Center at Peking University [65]. The index is constructed from indicators such as the breadth of digital financial coverage, the depth of digital financial usage, and digital inclusion, making it a representative measure of the digital financial environment in the provinces where banks are located.

This paper introduces an interaction term between DT and the standardized Digital Financial Inclusion Index (z1) into Equation (1). As shown in Column (1) of Table 7, the regression coefficient of the interaction term is significantly positive. This result indicates that there is heterogeneity in the digital financial development environment regarding the impact of digital transformation on return on assets. Specifically, when the main regression outcome is negative, the higher the level of digital financial development in the region, the greater the mitigating effect on the negative impact of digital transformation on return on assets.

**Table 7:** Heterogeneity analysis.

	(1)	(2)
	roa	roa
DT	−0.011^***^	−0.041^***^
	(0.003)	(0.013)
z1	0.288^***^	
	(0.106)	
DT*z1	0.004^***^	
	(0.002)	
DT*LDR		0.045^***^
		(0.017)
size	0.027	0.026
	(0.024)	(0.024)
LDR	−0.304^***^	−0.632^***^
	(0.101)	(0.188)
ProGDPr	0.023^***^	0.024^***^
	(0.006)	(0.006)
TD	−0.093^**^	−0.057
	(0.036)	(0.035)
TL	−0.168^***^	−0.171^**^
	(0.064)	(0.068)
cra	−0.735^***^	−0.613^**^
	(0.246)	(0.235)
pr	0.009^**^	0.012^***^
	(0.004)	(0.004)
_cons	1.495^**^	1.043
	(0.622)	(0.638)
*N*	1219	1219
R^2^	0.619	0.614
Year	YES	YES
Id	YES	YES

Standard errors in parentheses.

* *p* < 0.10, ** *p* < 0.05, *** *p* < 0.01.

The rationale is that a higher digital financial inclusion index implies a more advanced level of digitization in the region, more mature digital technology, and greater awareness among employees regarding digital transformation. The development of digital financial inclusion promotes the deepening of financial services by applying technologies such as big data and artificial intelligence in the financial sector, thereby improving financial service efficiency and addressing issues like information asymmetry [18] and risk identification.

As the level of digital financial development in a region improves, it can address the information asymmetry and bank-enterprise interaction issues that traditional financial inclusion faces, enhancing both credit quality and efficiency [19]. Higher levels of digital financial development allow businesses to access convenient, safe financial services more easily [36], and the use of big data mining and cloud computing analytics further drives the digital transformation of traditional banks. Compared to traditional financial inclusion, digital financial inclusion expands the supply of financial products and services, effectively broadening the coverage of financial services [16].

In more digitized regions, both banks and their employees are better equipped to adapt to digital transformation and apply the related technologies more efficiently, thereby offsetting the adverse effects of digital transformation on profitability performance.

### 6.2 Heterogeneity of loan-deposit ratio

Each bank’s loan-to-deposit ratio (LDR) varies, and this ratio is closely related to the bank’s asset-liability structure. To analyze the heterogeneity of LDR, this paper introduces an interaction term between digital transformation (DT) and the loan-to-deposit ratio (LDR) into Equation (2). The results in Column (2) of Table 7 show that the regression coefficient of the interaction term is significantly positive. This indicates that there is heterogeneity in the loan-to-deposit ratio regarding the impact of digital transformation on return on assets.

In this case, when the main regression outcome is negative, a higher loan-to-deposit ratio strengthens the bank’s ability to mitigate the negative impact of digital transformation on return on assets. A higher loan-to-deposit ratio implies that the bank pays relatively less interest on deposits and has lower capital costs, which helps to offset the negative effects of digital transformation on profitability performance.

## 7. Research conclusions and implications

### 7.1 Research findings

Drawing on panel data spanning from 2011 to 2023, encompassing 116 commercial banks, this study employs regression analysis to scrutinize the distinct characteristics and the intricate underlying mechanisms through which digital transformation influences bank profitability performance. The empirical findings are as follows:

Firstly, the results of the baseline regression analysis demonstrate that, during the study period, digital transformation has failed to bolster the profitability performance of banks. On the contrary, it has exerted a detrimental impact on their profitability performance.

Second, the mechanism analysis reveals that digital transformation negatively influences both operational efficiency and the non-performing loan ratio. Operational efficiency and the non-performing loan ratio act as mediating variables, transmitting the negative impact of digital transformation on return on assets. As banks undergo digital transformation, reductions in operational efficiency and increases in non-performing loans lead to lower returns on total assets, thus negatively affecting profitability performance.

Third, the digital financial inclusion environment and the loan-to-deposit ratio exhibit heterogeneous effects on the relationship between digital transformation and profitability performance. This indicates that the impact of digital transformation on profitability performance varies depending on the regional digital financial environment and the bank’s loan-to-deposit ratio.

### 7.2 Theoretical contributions

First, over the past decade, digital technologies have reshaped the banking industry, breaking down traditional barriers of time and space while introducing both opportunities and challenges. With the rapid adoption of big data, cloud computing, and artificial intelligence, digital transformation has become an inevitable strategic shift for banks worldwide. While originally aimed at enhancing profitability performance, the complexity, systemic nature, and high costs of digital transformation introduce uncertainty in its economic outcomes. The significant resource investments, opportunity costs, and steep learning curves in the early stages may negatively affect bank profitability performance [66]. This paper provides a comprehensive analysis of how digital transformation influences bank profitability performance, particularly during its early adoption phase. By examining mediating effects and heterogeneity, this research expands the discussion on performance determinants and finds that, from 2011 to 2023, digital transformation had an overall negative impact on bank profitability performance.

Second, extensive literature highlights the positive effects of fintech innovations in improving bank efficiency [67] and mitigating information asymmetry [68]. This paper contributes to this discussion by empirically validating the mediating roles of operational efficiency and the non-performing loan ratio in the relationship between digital transformation and bank profitability performance. The regression analysis identifies boundary effects that shape how digital transformation influences efficiency and credit risk. While prior research suggests that emerging technologies generate positive spillover effects in banking [67], this paper finds that such benefits have not yet fully materialized, particularly in banking sectors at an early stage of digital adoption. This provides a critical counterpoint to existing literature, encouraging scholars to reconsider the transitional challenges of digital transformation rather than assuming an immediate positive impact.

Third, research on spatial proximity in banking suggests that geographic closeness enhances access to credit [69,70,71,72]. However, some scholars argue that geographic factors are becoming less relevant in modern financial systems [73]. Addressing these contrasting views, this paper introduces a heterogeneity analysis of the Digital Financial Inclusion Index [65], moving beyond prior research that treats the index as a static variable. This approach provides a broader perspective on how the regional digital financial landscape influences bank profitability performance, demonstrating that geographic factors still play a role in digital banking transformation. This insight contributes to the global discourse on regional disparities in digital finance adoption, making the findings relevant beyond any single country or financial system.

### 7.3 Practical implications

First, it is important to take a balanced and objective view of the impact of digital transformation on the profitability performance of the banking sector. Although digital transformation is a clear trend in China’s banking industry, with significant annual investments, the research results suggest that banks have not escaped the “Productivity Paradox” when it comes to IT investment. As a traditional industry, the banking sector, both in terms of business types and workforce, requires more time to fully embrace and implement digital transformation. Since digital development in China’s banking industry is still in its early stages, the long-term outlook for improving quality and efficiency remains optimistic.

Second, banks should be encouraged to leverage digital transformation and actively adopt fintech to enhance risk management and improve the quality of credit assets. Commercial banks need to intensify their efforts in building fintech capabilities, particularly in the credit services domain. This includes establishing robust mechanisms for recruiting, training, and incentivizing tech talent and integrating advanced technologies into the credit business management process. Employees should be encouraged to use cutting-edge technology to manage credit risk, reducing information asymmetry between banks and firms, and optimizing risk prevention and control.

Third, the banking industry should accelerate the research and application of fintech. As new generations of information technology rapidly evolve, fintech is becoming a key component of the digital age. Banks must organically integrate fintech with digital transformation, driving both technology-driven and digitally-driven innovations. Local governments should implement national digital economy development strategies by enhancing the supply of digital economy policies. They should also foster collaboration between local banks and external tech companies, encouraging fintech firms to introduce new technologies into the banking sector.

### 7.4 Limitations and future outlook

This paper explores the impact of digital transformation on the profitability performance of banks. In this paper, return on assets (ROA) is used as the dependent variable for the regression analysis, but there is room to expand the dimensions for measuring profitability performance to make the regression analysis more comprehensive. The specific recommendations are as follows:

First, additional financial indicators could be used as dependent variables to measure profitability performance, such as operating income or the natural logarithm of net profit, to assess whether digital transformation negatively impacts multiple performance measures simultaneously. Additionally, digital transformation may reduce the communication and interaction time between banks and customers, and the shift from paper-based to online processes could also improve employee productivity. To fully capture the impact on profitability performance, it may be useful to define bank performance by using total factor productivity (TFP) as the dependent variable to assess the impact of digital transformation through regression analysis.

Second, there are limitations regarding data collection and processing in this paper. The data used for the 2011–2023 period were compiled from large databases and annual reports disclosed by banks. However, some banks did not disclose all relevant indicators in the earlier years, making it difficult to collect complete and high-quality data. To fill in the gaps for unavailable data, this paper employed interpolation and averaging methods, which may affect the statistical significance and generalizability of the results. As time progresses and digital transformation deepens, banks are likely to disclose more transparent and accessible data, which will facilitate future research on the impact of digital transformation. Additionally, methods like web scraping could be used to gather more comprehensive data, further improving the robustness and completeness of future research results.

Third, a nonlinear (U-shaped) relationship between digital transformation (DT) and bank profitability (ROA) is theoretically plausible. However, the current sample period (2011–2023) corresponds to the early stage of DT adoption in China’s banking sector, limiting the ability to capture the right-hand, long-run recovery segment of this potential curve. Future research with extended time horizons will be better positioned to test nonlinear dynamics and long-term performance effects.
